# Correction: Hashmi et al. Hydrogen Sulphide Treatment Prevents Renal Ischemia-Reperfusion Injury by Inhibiting the Expression of ICAM-1 and NF-kB Concentration in Normotensive and Hypertensive Rats. *Biomolecules* 2021, *11*, 1549

**DOI:** 10.3390/biom12040593

**Published:** 2022-04-18

**Authors:** Syed F. Hashmi, Hassaan Anwer Rathore, Munavvar A. Sattar, Edward J. Johns, Chee-Yuen Gan, Tan Yong Chia, Ashfaq Ahmad

**Affiliations:** 1 School of Pharmaceutical Sciences, Universiti Sains Malaysia, Penang 11800, Malaysia; fayazhashmi84@gmail.com (S.F.H.); hrathore@qu.edu.qa (H.A.R.); munavvar@usm.my (M.A.S.); 2 Department of Physiology, University College Cork, T12 K8AF Cork, Ireland; ej.johns@ucc.ie; 3 Analytical Biochemistry Research Centre (ABrC), Universiti Sains Malaysia (USM), Lebuh Bukit Jambul, Penang 11900, Malaysia; cygan@usm.my; 4 Department of Pharmacy Practice, College of Pharmacy, University of Hafr Al-Batin, Hafr Al-Batin 31991, Saudi Arabia

In the published publication [1], there was an error regarding the affiliation for Hassaan Anwer Rathore. In addition to affiliation 1, the current address should be: College of Pharmacy, Qatar University, Doha P.O. Box 2713, Qatar. The authors apologize for any inconvenience caused and state that the scientific conclusions are unaffected. This correction was approved by the Academic Editor. The original publication has also been updated.

## References

[1] Hashmi S.F., Rathore H.A., Sattar M.A., Johns E.J., Gan C.Y., Chia T.Y., Ahmad A. (2021). Hydrogen Sulphide Treatment Prevents Renal Ischemia-Reperfusion Injury by Inhibiting the Expression of ICAM-1 and NF-kB Concentration in Normotensive and Hypertensive Rats. Biomolecules.

